# Visualization of two-dimensional transverse blood flow direction using optical coherence tomography angiography

**DOI:** 10.1117/1.JBO.25.12.126003

**Published:** 2020-12-16

**Authors:** Inho Shin, Wang-Yuhl Oh

**Affiliations:** a Korea Advanced Institute of Science and Technology, Department of Mechanical Engineering, Daejeon, Republic of Korea; b Korea Advanced Institute of Science and Technology, KI for Health Science and Technology, Daejeon, Republic of Korea

**Keywords:** biomedical optics, optical coherence tomography angiography, two-dimensional flow direction

## Abstract

**Significance:** Evaluation of vessel patency and blood flow direction is important in various medical situations, including diagnosis and monitoring of ischemic diseases, and image-guided vascular surgeries. While optical coherence tomography angiography (OCTA) is the most widely used functional extension of optical coherence tomography that visualizes three-dimensional vasculature, inability to provide information of blood flow direction is one of its limitations.

**Aim:** We demonstrate two-dimensional (2D) transverse blood flow direction imaging in *en face* OCTA.

**Approach:** A series of triangular beam scans for the fast axis was implemented in the horizontal direction for the first volume scan and in the vertical direction for the following volume scan, and the inter A-line OCTA was performed for the blood flow direction imaging while the stepwise pattern was used for each slow axis scan. The decorrelation differences between the forward and the backward inter A-line OCTA were calculated for the horizontal and the vertical fast axis scans, and the ratio of the horizontal and the vertical decorrelation differences was utilized to show the 2D transverse flow direction information.

**Results:** OCTA flow direction imaging was verified using flow phantoms with various flow orientations and speeds, and we identified the flow speed range relative to the scan speed for reliable flow direction measurement. We demonstrated the visualization of 2D transverse blood flow orientations in mouse brain vascular networks *in vivo*.

**Conclusions:** The proposed OCTA imaging technique that provides information of 2D transverse flow direction can be utilized in various clinical applications and preclinical studies.

## 1 Introduction

Assessment of vessel patency and blood flow direction is critical in various medical situations, including diagnosis and treatment monitoring of ischemic diseases^1^^–^^8^ and image-guided vascular surgeries.^9^^–^^21^ Ischemic diseases are typically characterized by the disruption of the blood flow, such as changes in blood flow speed and blood flow direction.^11^ In the treatment of ischemic diseases, the natural and procedurally promoted vascular remodeling of existing arterial interconnections to form adequate collateral development for restoration of perfusion to the organs that have suffered ischemia is closely related to the patient outcomes.^2^^–^^7^ Non-invasive monitoring of blood flow direction and occasional flow direction reversal in the treatment of ischemic diseases can provide important information to assess therapeutic effectiveness.^1^^–^^5^ In various vascular surgeries such as vascular bypass,^13^^–^^17^ vascular shunt,^5^^,^^18^^–^^21^ and aneurysm surgery,^9^^,^^10^^,^^14^^,^^17^ blood flow characteristics such as blood flow path and blood flow direction are intentionally modified. Imaging the blood flow direction during the surgeries that confirms the intended changes of the paths and directions of the blood flow while avoiding any unintended alteration in vessel patency and blood flow direction is highly desirable for monitoring of the immediate effects of the surgery and for quality control of the vascular intervention.^5^^,^^9^^,^^10^

While optical coherence tomography angiography (OCTA) is the most widely used functional form of optical coherence tomography (OCT) that visualizes three-dimensional vasculature without a need of exogenous contrast agent injection,^22^^–^^26^ inability to provide information of blood flow direction is one of its limitations. Doppler ultrasound^3^^–^^5^^,^^8^^–^^13^^,^^20^ and Doppler OCT^27^^–^^29^ have been main imaging tools used for measuring the flow direction. However, the Doppler techniques can only provide the axial flow direction, whether the flow moves toward or away from the imaging wave source, and therefore usually visualize the axial flow directions in cross-sectional images. Moreover, Doppler OCT typically requires additional phase stabilization or phase calibration because it is based on measurement of the Doppler phase difference. Laser speckle imaging and multiphoton laser scanning microscopy also showed flow direction imaging, but they required vessel by vessel scanning for tracking injected fluorescent-labeled red blood cells or fluorescent microbeads.^1^^,^^2^

In this work, we demonstrate an OCTA imaging technique for the visualization of two-dimensional (2D) transverse blood flow direction information. We implemented the inter A-line OCTA using an isosceles triangular fast axis beam scan and calculated the difference between OCTA decorrelation signals acquired from the forward and backward beam scan sections to determine the flow direction in the fast axis. Performing a pair of consecutive OCTA volume scans with the horizontal fast axis scan and the vertical fast axis scan, respectively, provided 2D transverse flow direction information. OCTA flow direction imaging was verified using flow phantoms with various orientations. We demonstrated the visualization of the blood flow orientations in mouse brain vascular networks *in vivo*.

## 2 Methods

**Fig. 1:**
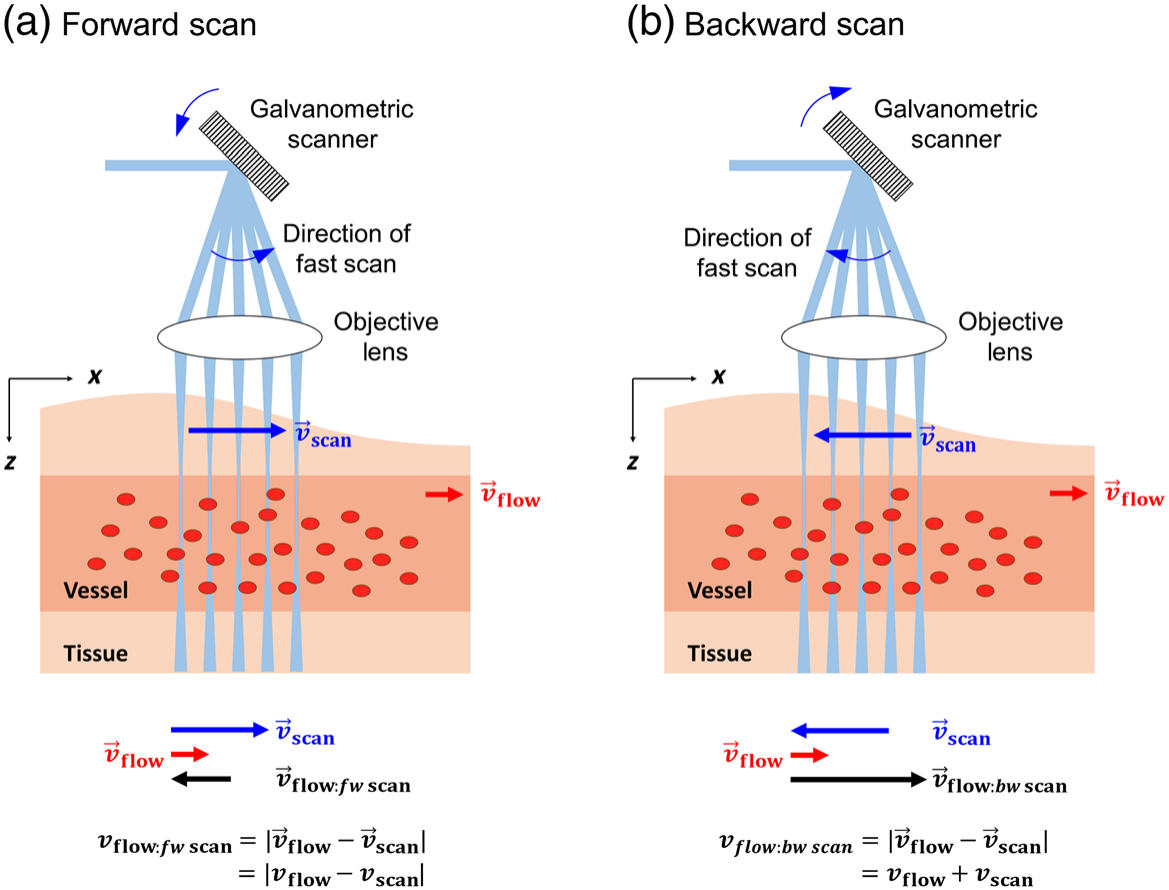
The relative blood flow speed with respect to the imaging beam scan speed with (a) forward scan (imaging beam scans in the same direction of the flow) and (b) backward scan (imaging beam scans in the opposite direction of the flow).

In OCTA, the magnitude of the decorrelation signal is proportional to the displacement of the scatterers in the flow during the time interval between two measurements when the imaging beam is at the same location for the two measurements.^30^^–^^32^ When the imaging beam moves between the two measurements, the inter A-line OCTA decorrelation signal is determined by the relative displacement of the scatterers with respect to the location of the imaging beam in each of the two measurements. For the same flow, the effective displacement of the scatterers becomes smaller when the imaging beam scans in the same direction of the flow than when it scans in the opposite direction of the flow as depicted in Fig. 1. Therefore, the decorrelation signal is also measured as a smaller value when the imaging beam scans in the same direction of the flow than when it scans in the opposite direction of the flow, and the one-dimensional flow direction information can be obtained by comparing the OCTA signals acquired during the forward and the backward imaging beam scans. The 2D flow direction information is obtained by performing a pair of the inter A-line OCTA volume scans with the fast axis scans in the horizontal direction and the vertical direction, respectively.

**Fig. 2:**
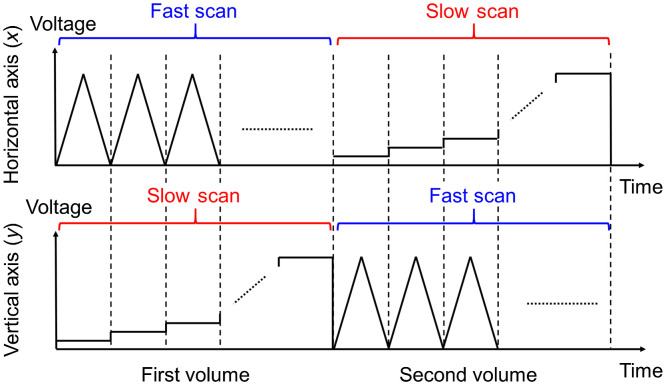
Beam scanning protocol of the inter A-line OCTA for 2D transverse flow direction imaging.

An OCTA system with a lab-built short cavity wavelength-swept laser operating at 1300 nm as a light source was used in this work.^33^^,^^34^ The axial and transverse resolutions were measured to be 11.3 and 8.2  μm in air, respectively, and the system sensitivity was measured to be 102 dB at an A-line rate of 220.4 kHz. Figure 2 illustrates the beam scanning protocol for flow direction OCTA imaging. It consists of a pair of consecutive inter A-line OCTA volume scans. A volume scan with horizontal direction as the fast axis is performed, followed by another volume scan with vertical direction as the fast axis. At each B-scan position in the vertical direction, an isosceles triangular scan was performed to provide both forward and backward fast axis scans with the same scan speed. Upon completion of each isosceles triangular scan, the imaging beam was subsequently stepped to the next B-scan position. The same volume scan is repeated but with the fast axis isosceles triangular scan in the vertical direction and the slow axis stepwise scan in the horizontal direction.

The decorrelation signal from the inter A-line OCTA is proportional to the displacement of the scatterers in the flow during the inter A-line time interval and can be expressed as a linear function of the displacement, such as D=α×Δx=α×v×Δt, when the displacement is smaller than approximately 22% of the resolution,^31^ where D is the decorrelation,^32^ Δx is the displacement of moving scatterers in the flow, α is a proportional coefficient, v is the flow speed relative to the beam scan speed, and Δt is the inter A-line time interval. With the horizontal (x) fast axis scan, the decorrelation signal can be expressed as $D_{x,f}=\alpha \Delta t|v_{\text{flow}}\cos\theta -v_{\text{scan}}|+D_{o}$ for the forward scan section and $D_{x,b}=\alpha \Delta t|v_{\text{flow}}\cos\theta +v_{\text{scan}}|+D_{o}$ for the backward scan section, where θ is the angle between the flow direction and the horizontal forward direction, $v_{\text{flow}}$ is the flow speed, $v_{\text{scan}}$ is the fast axis scan speed, and $D_{o}$ is the baseline decorrelation signal with no directional flow. If the scan speed is higher than the horizontal flow speed, the difference between decorrelation signals obtained from the forward scan and the backward scan is given by $\Delta D_{x}=D_{x,b}-D_{x,f}=2\alpha \Delta tv_{\text{flow}}\;\cos\;\theta$. With the vertical (y) fast axis scan, the decorrelation signal difference between the forward and backward OCTA can be expressed as $\Delta D_{y}=D_{y,b}-D_{y,f}=2\alpha \Delta tv_{\text{flow}}\;\sin\;\theta$. Therefore, the flow direction information can be obtained as θ=arctan ($\Delta D_{y}/\Delta D_{x}$).

## 3 Results and Discussions

**Fig. 3:**
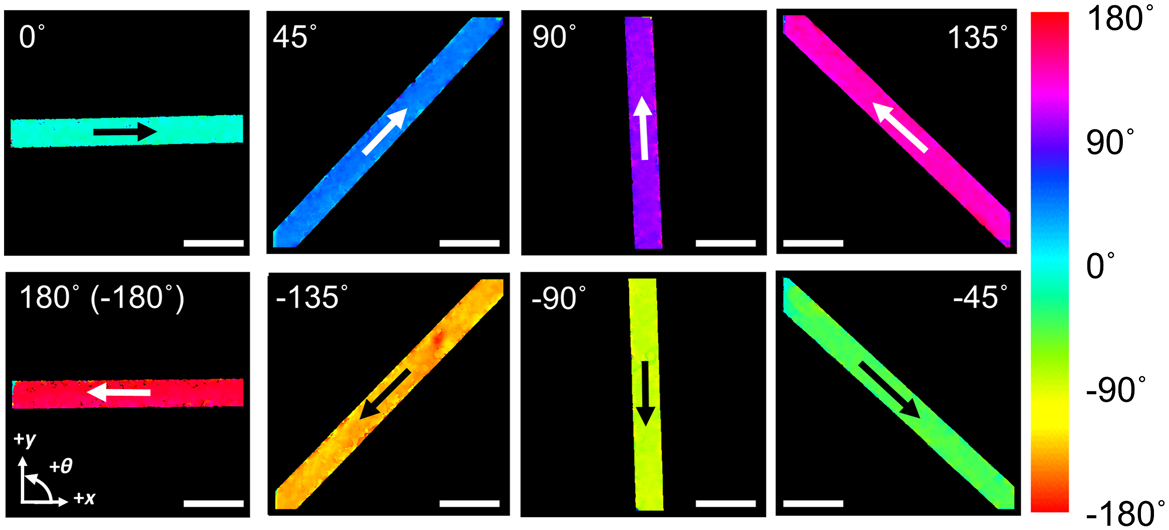
Flow phantom images showing transverse flow direction measurements. Arrows inside the tube flow phantom indicate the flow directions. Flow speed was set at 50 mm/s. Scale bar: 500  μm.

We first demonstrated the visualization of the 2D transverse flow direction using flow phantoms oriented in various angles. The flow phantom consisted of a transparent tube with an inner diameter of 150  μm (CAP360-150P, LabSmith) embedded in a polydimethylsiloxane block with ${\mathrm{TiO}}_{2}$ added as scattering particles. Milk was pumped using a syringe pump (NE-300, New Era Pump Systems, Inc.) to form flow in the tube. The average flow speed was set at 50 mm/s, and an area over 2  mm×2  mm was imaged with 2048 A-lines ×1024 B-scans. Figure 3 shows a clear visualization of the 2D transverse flow directions inside the tube phantoms oriented eight different directions with a 45-deg interval.

**Fig. 4:**
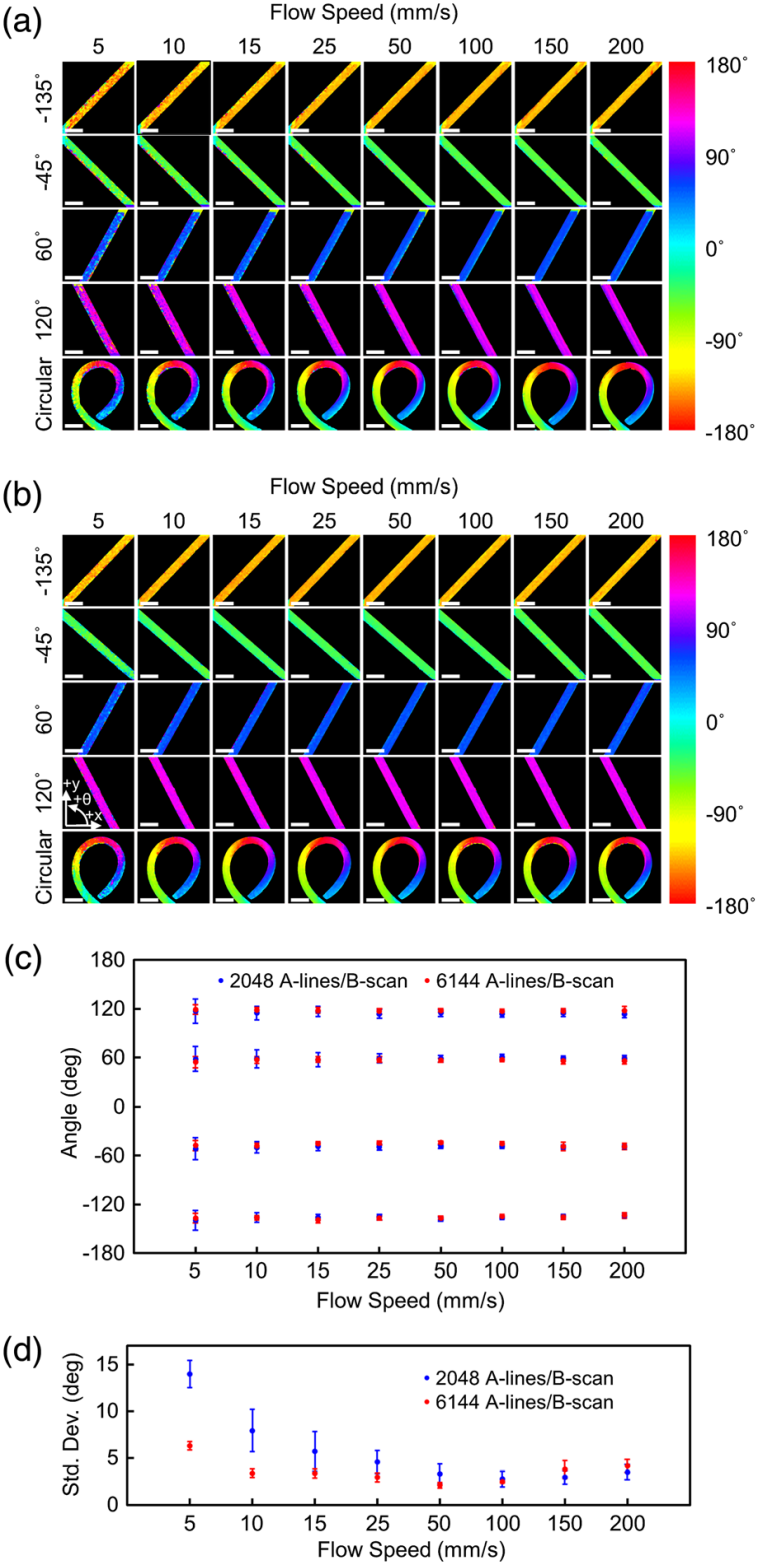
Flow phantom images over a 2  mm×2  mm area with various flow speeds ranging from 5 to 200 mm/s. (a) Images acquired using 2048 A-lines/B-scan. (b) Images acquired using 6144 A-lines/B-scan. (c) Mean flow directions measured with the linear flow phantoms at various flow speeds. (d) Standard deviations of the flow direction measurements for the linear flow phantoms at various flow speeds. Scale bar: 500  μm.

To identify the flow speed range that provides a reliable flow direction measurement, we imaged the flow phantom of various speeds ranging from 5 to 200 mm/s using two different fast axis scan speeds. Figures 4(a) and 4(b) show color-encoded transverse flow direction OCTA images of a 2  mm×2  mm area of the flow phantom acquired with 2048 A-lines/B-scan and 6144 A-lines/B-scan, which correspond to the fast axis scan speed of 215 and 71.7 mm/s, respectively, for four different linear flows and a circular flow with various speeds. Figures 4(c) and 4(d) show the measured mean flow directions and their standard deviations within the ROI of 120  μm width and 1.2 mm length along the center of the tube for the four linear flow phantoms. The flow directions were well-determined with the standard deviations smaller than 5 deg when the flows were faster than 22 mm/s with 2048 A-lines/B-scan ($v_{\text{scan}}=215\;\mathrm{mm}/s$) and 7 mm/s with 6144 A-lines/B-scan ($v_{\text{scan}}=71.7\;\mathrm{mm}/s$), respectively, which approximately correspond to 10% of the beam scan speed. As the flow direction is determined by the difference in relative flow speeds with respect to the beam scan speeds acquired during the forward and backward beam scan periods, the accurate flow direction measurement is hampered when the flow speed is too small compared to the scan speed. To maintain accurate flow direction measurements for slow flows, the scan speed needs to be slowed down by oversampling at the expense of the imaging speed.

It is also noteworthy that the standard deviation of the flow direction measurement was the minimum at a flow speed of approximately 50 mm/s and increases as the flow speed increases with the fast axis scan speed of 71.7 mm/s (6144 A-lines/B-scan). For the fast axis scan speed of 215 mm/s (2048 A-lines/B-scan), the standard deviation of the flow direction measurement monotonically decreased to a flow speed of 150 mm/s, but a slight increase was observed as the flow speed reached approximately 200 mm/s. For the determination of the flow direction information, we assumed that the fast axis scan speed is higher than the flow speed component parallel to the fast axis. While the fast axis scan speed is always higher than the fastest flow speed used in the experiment with the fast axis scan speed of 215 mm/s, the flow speed component parallel to the fast axis can be higher than the scan speed with the fast axis scan speed of 71.7 mm/s (6144 A-lines/B-scan), which contributed the increased standard deviation of the flow direction measurement at the higher flow speeds when imaging with 6144 A-lines/B-scan.

**Fig. 5:**
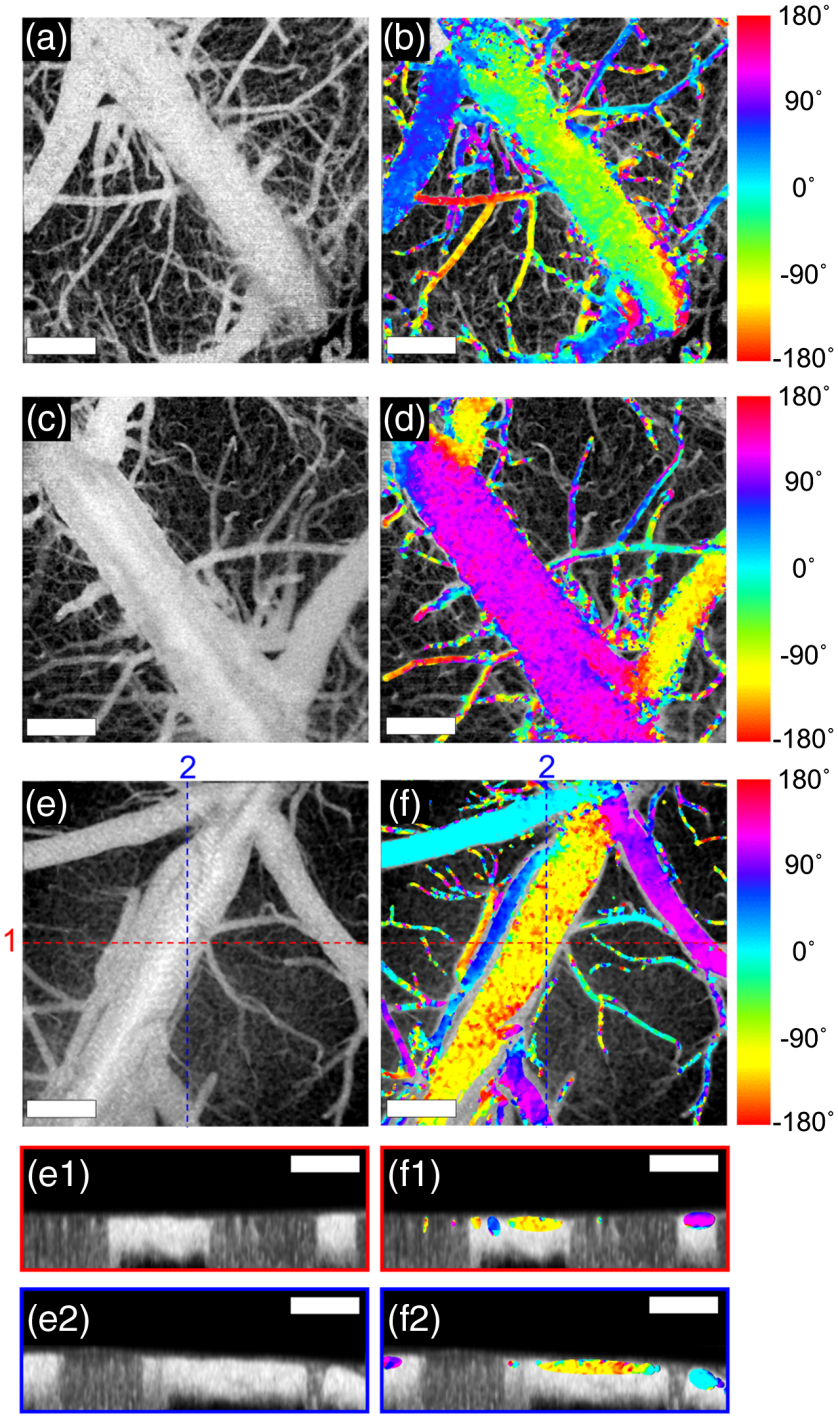
*In vivo* 2D transverse flow direction images of mouse brains. (a), (c), and (e) *En face* mean projections of the inter B-scan OCTA volume images, and (b), (d), and (f) color-encoded blood flow direction information overlaid on the corresponding *en face* OCTA images. (e1), (e2), (f1), and (f2) Cross-sectional OCTA images and color-encoded blood flow direction images at locations indicated by dashed lines in the *en face* OCTA images. Scale bar: 500  μm

*In vivo* OCTA imaging of blood flow direction was demonstrated in the cranial windows implanted on the mouse brains. The animal experiments were performed under the approval of the Institutional Animal Care and Use Committee of Korea Advanced Institute of Science and Technology (KAIST).^35^ Male C57BL/6 mice (>8 weeks) were anesthetized with 1.5% to 3% (v/v) of isoflurane during surgical and imaging procedures. The head of a mouse was fixed in a stereotaxic frame and a circular cranial window was implanted on the cortex for imaging. The mouse brain over a field of 2  mm×2  mm (6144 A-lines ×1024 B-scans) was imaged with the proposed beam scanning protocol for the flow direction imaging. Figures 5(a), 5(c), and 5(e) show the *en face* mean projections of the inter B-scan OCTA volume images. Figure 5(c) shows the *en face* OCTA image acquired by rotating the same mouse cranial window used for the imaging of Fig. 5(a) by 180 deg. Figure 5(e) shows a cranial window OCTA image of another mouse. In Figs. 5(b), 5(d), and 5(f), the color-encoded information of blood flow directions was overlaid on the *en face* OCTA images. Cross-sectional OCTA images and color-encoded blood flow direction images at locations indicated by dashed lines in the *en face* OCTA images are shown in Figs. 5(e1), 5(e2), 5(f1), and 5(f2). The blood flow directions in relatively large vessels with the blood flow speed higher than the slowest direction-detectable flow speed, which is approximately 7 mm/s in the image with 6144 A-lines×1024 B-scans for a 2  mm×2  mm area, were well visualized. Flow directions in the capillaries were not detected because the blood flow speed in most capillaries is lower than the slowest direction-detectable flow speed. While the direction of the slow blood flows in capillaries may be detected by decreasing the fast axis scan speed through dense oversampling, reliable flow direction measurements can be limited for flows faster than the fast axis scan speed.

## 4 Conclusion

We demonstrate an OCTA imaging technique that provides information of 2D transverse flow direction. Implementation of a pair of consecutive inter A-line OCTA volume scans using triangular fast axis beam scans in the horizontal direction for the first volume and in the vertical direction for the following volume, respectively, enabled determination of the 2D transverse flow direction through calculation of difference between OCTA decorrelation signals acquired during the forward and backward fast axis beam scans. Through flow phantom experiments, we verified the OCTA flow direction imaging and identified the flow speed range relative to the scan speed for reliable flow direction measurement. *In vivo* OCTA for 2D transverse blood flow direction was presented in the mouse cranial window imaging. The proposed OCTA imaging technique may be very useful in various applications where the assessment of the transverse blood flow direction information is important such as diagnosis and treatment monitoring of ischemic diseases, image-guided vascular surgeries, and preclinical studies using animal vascular disease models.
